# Antepartum Membrane Stripping in GBS Carriers, Is It Safe? (The STRIP-G Study)

**DOI:** 10.1371/journal.pone.0145905

**Published:** 2015-12-31

**Authors:** Doron Kabiri, Yael Hants, Tom Raz Yarkoni, Esther Shaulof, Smadar Eventov Friedman, Ora Paltiel, Ran Nir-Paz, Wesam E. Aljamal, Yossef Ezra

**Affiliations:** 1 Department of Obstetrics and Gynecology, Hadassah-Hebrew University Medical Center, Jerusalem, Israel; 2 Department of Neonatology, Hadassah-Hebrew University Medical Center, Jerusalem, Israel; 3 Braun School of Public Health and Community Medicine, Hadassah-Hebrew University Medical Center, Jerusalem, Israel; 4 Department of Clinical Microbiology and Infectious Diseases, Hadassah-Hebrew University Medical Center, Jerusalem, Israel; The University of Hong Kong, HONG KONG

## Abstract

**Objective:**

Stripping of the membranes is an established and widely utilized obstetric procedure associated with higher spontaneous vaginal delivery rates, reduced need for formal induction of labor and a lower likelihood of post-term pregnancy. Due to the theoretical concern of bacterial seeding during the procedure many practitioners choose not to sweep the membranes in Group B Streptococcus (GBS) colonized patients. We conducted ‘the STRIP-G study’ in order to determine whether maternal and neonatal outcomes are affected by GBS carrier status in women undergoing membrane stripping.

**Study design:**

We conducted a prospective study in a tertiary referral center, comparing maternal and neonatal outcomes following membrane stripping among GBS-positive, GBS-negative, and GBS-unknown patients. We compared the incidence of composite adverse neonatal outcomes (primary outcome) among the three study groups, while secondary outcome measure was composite adverse maternal outcomes.

**Results:**

A total of 542 women were included in the study, of which 135 were GBS-positive, 361 GBS-negative, and 46 GBS-unknown status. Demographic, obstetric, and intra-partum characteristics were similar for all groups. Adverse neonatal outcomes were observed in 8 (5.9%), 31 (8.6%), and 2 (4.3%) in the GBS-positive, GBS-negative, and unknown groups, respectively (P = 0.530), (Odds Ratio between GBS-Positive vs. GBS-Negative groups 0.67 (95%, CI = 0.30–1.50)); while composite adverse maternal outcomes occurred in 9 (6.66%), 31 (8.59%), and 5 (10.87%) in the GBS-positive, GBS-negative, and unknown groups, respectively (P = 0.617).

**Conclusions:**

Antepartum membrane stripping in GBS carriers appears to be a safe obstetrical procedure that does not adversely affect maternal or neonatal outcomes.

## Introduction

Stripping or sweeping of the fetal membranes is a widely utilized technique to hasten delivery, first described by James Hamilton in 1810 [1, 2]. The procedure is performed during a vaginal examination, by separating the chorioamniotic membrane from the lower uterine segment by a circular movement of the finger. This maneuver is thought to initiate a cascade of physiological reactions in which local production of prostaglandins promotes cervical ripening and may lead to the onset of active labor [3]. Several studies have reported that membrane stripping is associated with higher rates of spontaneous vaginal delivery, shorter induction-to-delivery interval, reduced likelihood of post-term pregnancy, and a decrease in the need for induction of labor [3–6]. Adverse effects commonly reported following the procedure are limited to transient maternal discomfort during the procedure, irregular uterine contractions, and clinically insignificant vaginal bleeding.

*Streptococcus agalactiae* (Group B Streptococcus (GBS)) is the leading major cause of early neonatal sepsis and an important pathogen associate with maternal peripartum infection [7]. Early onset neonatal GBS disease, defined as infection presenting in the first 7 days of life, has a broad clinical spectrum of ranging from mild illness that resolves spontaneously within several days, to severe respiratory disease, meningitis, neonatal sepsis, and death [7, 8]. It is estimated that 20–30% of all pregnant women are colonized with GBS [9].

A recent Cochrane review confirmed that membrane stripping does not increase the risk of maternal and neonatal infection, however this review did not analyze the outcomes by GBS carrier state [10]. However, based on the theoretical increased risk of bacterial seeding, as well as concern for fast labor which would prevent the administration of adequate antibiotic prophylaxis after membrane stripping, some practitioners choose not to sweep the membranes in GBS colonized patients. Both the American College of Obstetricians and Gynecologists (ACOG) and the Centers for Disease Control (CDC) do not consider GBS colonization as a contraindication to membrane stripping. Their latest guidelines discuss the risks of membrane stripping in women colonized with GBS. They concluded that the subject has not been investigated in well-designed prospective studies and therefore data are insufficient to encourage or discourage this practice in women known to be GBS-colonized [11–13].

Given the rare neonatal morbidity caused by GBS sepsis [14] as well as the lack of well-powered studies designed to address the safety of membrane stripping in known GBS carriers [15], approaches are inconsistent, [16, 17], and consensus has not been reached regarding whether antepartum membrane stripping in GBS carriers adversely affects maternal or neonatal outcomes.

In order to further elucidate the effect of antepartum membrane stripping in GBS carriers, we conducted a prospective study, the STRIP-G study, which examined whether the rates of adverse neonatal and maternal outcomes differ by GBS carrier status among women undergoing membrane stripping.

## Methods

### Study Design and participants

We conducted a prospective observational cohort study in the maternal-fetal unit of a tertiary center university teaching hospital with approximately 5,000 deliveries per year. The study population consisted of all women who underwent membrane stripping at this hospital between October 10, 2011 and December 31, 2013. All candidates for vaginal delivery with a singleton pregnancy and confirmed cephalic presentation between 37 0/7 and 41 6/7 weeks' gestational age were eligible. We excluded from this study women with a closed cervix, multiple gestation pregnancies, pregnancies with major fetal anomalies, and those who were not candidates for vaginal delivery (placenta previa, breech presentation, planned cesarean delivery). GBS-positive women were considered “exposed”, and the comparison groups consisted of women who were GBS-screening negative and those with unknown-GBS status. Detailed demographic data and medical, prenatal and antenatal history, was extracted by trained staff using the patients' electronic medical records. Postpartum, maternal and neonatal outcomes were collected along with maternal and newborn discharge summaries by trained study investigators.

The Hadassah Medical Organization Institutional Review Board approved the STRIP-G study and waived the requirement for informed consent (IRB N°: 0204-11-HMO). The obstetrical staff performed membrane stripping after explanation and obtaining patient’s consent to the procedure. Since the IRB considered this as an observational study, written informed consent was not required.

### Treatment Protocol

During the study period and in the absence of contraindications, women anticipating vaginal delivery were offered membrane stripping. In our institution we followed the RCOG and NICE guidelines for membrane stripping [18, 19] offering membrane stripping to all women prior to formal induction of labor, to nulliparous women at 40 weeks, to all women at the 41 week antenatal visit, and whenever the cervix assessed at term. No further restrictions or risk-based selection was done prior to offering membrane stripping. The obstetrical staff, physicians or midwives performed stripping after explanation and obtaining patient’s consent to the procedure. An ultrasonographic scan was performed before the procedure to verify fetal presentation and biophysical profile. In addition, prior to the procedure, fetal heart rate tracing (for nonstress testing) was obtained to confirm fetal well-being. After membrane stripping, women were discharged for routine obstetric care according to a standard local protocol, until the onset of spontaneous labor. Induction of labor was performed if the participant reached 42 weeks’ gestation or earlier if there was any neonatal or maternal indication that justified induction of labor.

Screening for GBS is routinely offered as part of obstetrical surveillance. According to our protocol, intrapartum antibiotic prophylaxis (IAP) was administered to women with known vaginal/intestinal tract GBS colonization, documented urinary tract infection with GBS at any time during the present pregnancy, a history of an infant with GBS disease in any prior delivery, or unknown GBS status with ruptured membranes for 18 hours or more. For every woman with suspected chorioamnionitis (defined as intrapartum maternal fever greater than 38°C or signs of chorioamnionitis), blood and urine cultures were taken and broad-spectrum intravenous antibiotics were administrated, including coverage for group B streptococcus.

### Outcomes

The primary outcome measure was incidence of composite adverse neonatal outcomes based on the NICE criteria for early detection of neonatal sepsis [20], while secondary outcome measure was composite adverse maternal outcomes.

**Fig 1:**
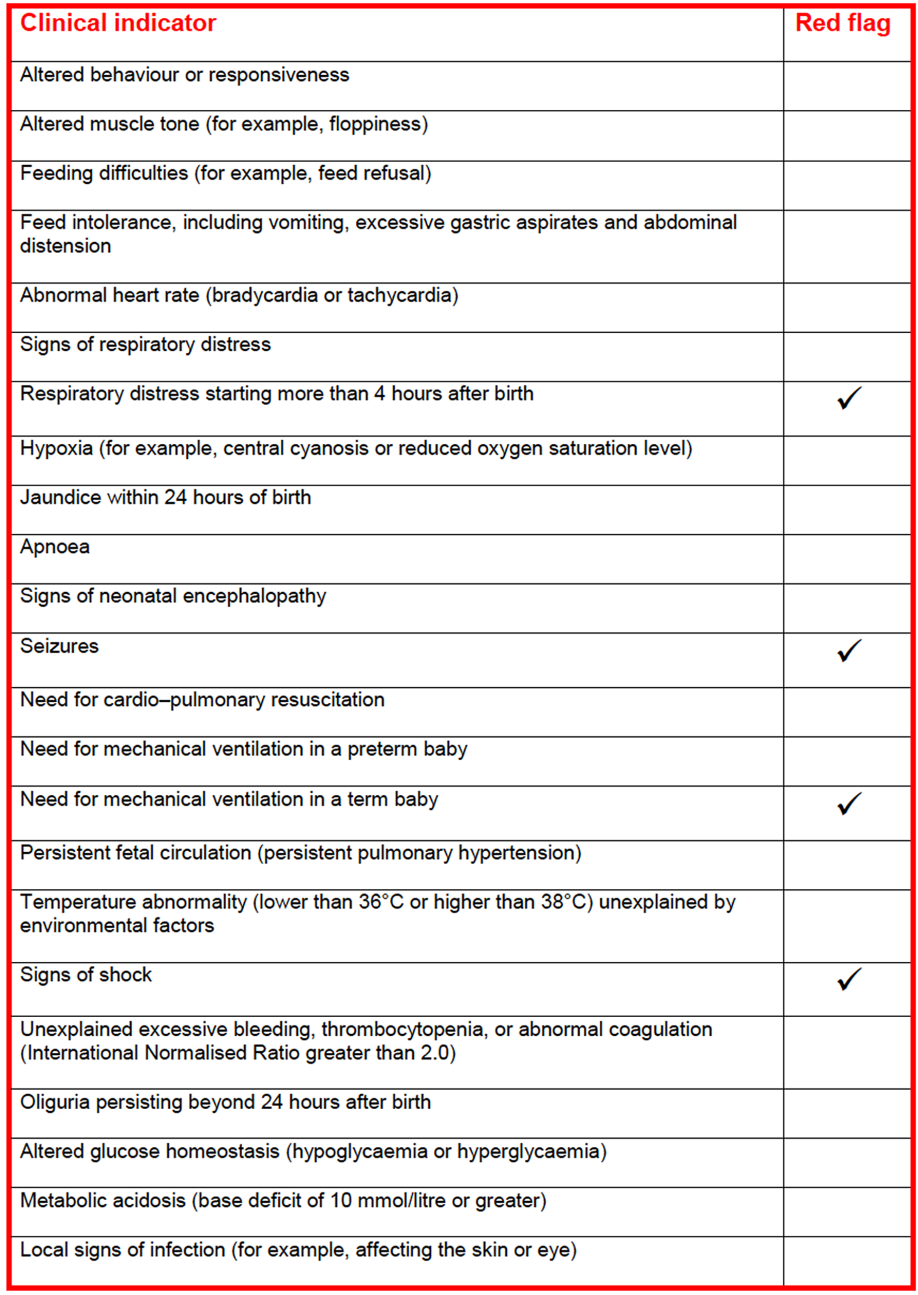
Clinical Indicators Of Possible-Early-Onset Neonatal Infection. (Figure reproduced from “Antibiotics for early-onset neonatal infection”, NICE clinical guidelines 149, 2012).

We did not choose early-onset neonatal GBS sepsis as the primary outcomes because the rarity of this event (incidence of 0.17–0.3 cases per 1000 births), that renders the assumption of bacterial seeding during membrane stripping difficult to demonstrate, and it is almost impossible to design a well-powered study that would be able to achieve statistical significance by using neonatal GBS sepsis as a primary endpoint. Following the assumption that a healthy neonate would not progress directly from a perfect state of health to full blown neonatal sepsis, the STRIP-G specialist group (which includes expert in perinatology, neonatology, microbiology and epidemiology), constructed a formal and structured scale aiming to detect a “compromised neonate” as a surrogate primary endpoint, based on the NICE criteria for early detection of neonatal sepsis [20]. The NICE criteria are produced by the National Institute for Health and Care Excellence in the United Kingdom to guide practitioners regarding which neonates should receive antibiotics for suspected early-onset GBS infection. According to the NICE guidelines, clinical indicators of possible early-onset neonatal infection include 19 clinical indicators and 4 ‘red flags’ indicators (Fig 1). Therefore, we performed a systematic review of medical records for each neonate whose mother participated in the study in order to identify any “compromised neonate”, defined as the presence of at least one ‘red flag’ or two clinical indicators of possible early-onset neonatal infection [20].

Adverse maternal outcome was defined as one or more of the following: chorioamnionitis, suspected chorioamnionitis or endometritis (defined by peripartum maternal fever and uterine tenderness), urinary tract infection or sepsis in the peripartum period, prolonged hospital stay, and allergic reaction to antibiotics.

We collected peripartum data until discharge from the hospital, and compared maternal and neonatal adverse outcomes among GBS-screening positive group, GBS-screening negative group and GBS unknown group.

### Statistical methods

Statistical analysis was performed by using the SPSS package (version 19, SPSS Inc., Chicago, IL, USA). Differences in continuous variables among the three groups were analyzed by the ANOVA. Differences in categorical variables were analyzed via the χ2 or Fisher's exact test. A two-sided P<0.05 was considered statistically significant. Risk differences between GBS-Positive and GBS-Negative groups and their standard error as well as odds ratios were calculated in WinPepi **®**.

We based our statistical analysis on the primary outcome (compromised neonate). The sample size was thus based on the expected difference between the study and control groups in percentage of ‘compromised neonate’. For the assumption that the ratio between the size of the GBS-positive and the GBS-negative group would be 1:3, with significance level of 5% (2 sided), and the proportion of compromised neonatal rate in the population is 6–8%, we estimated that 125 GBS-Positive women and 375 GBS-Negative women would provide power of 80% to detect a risk ratio of 2.2.

## Results

**Fig 2:**
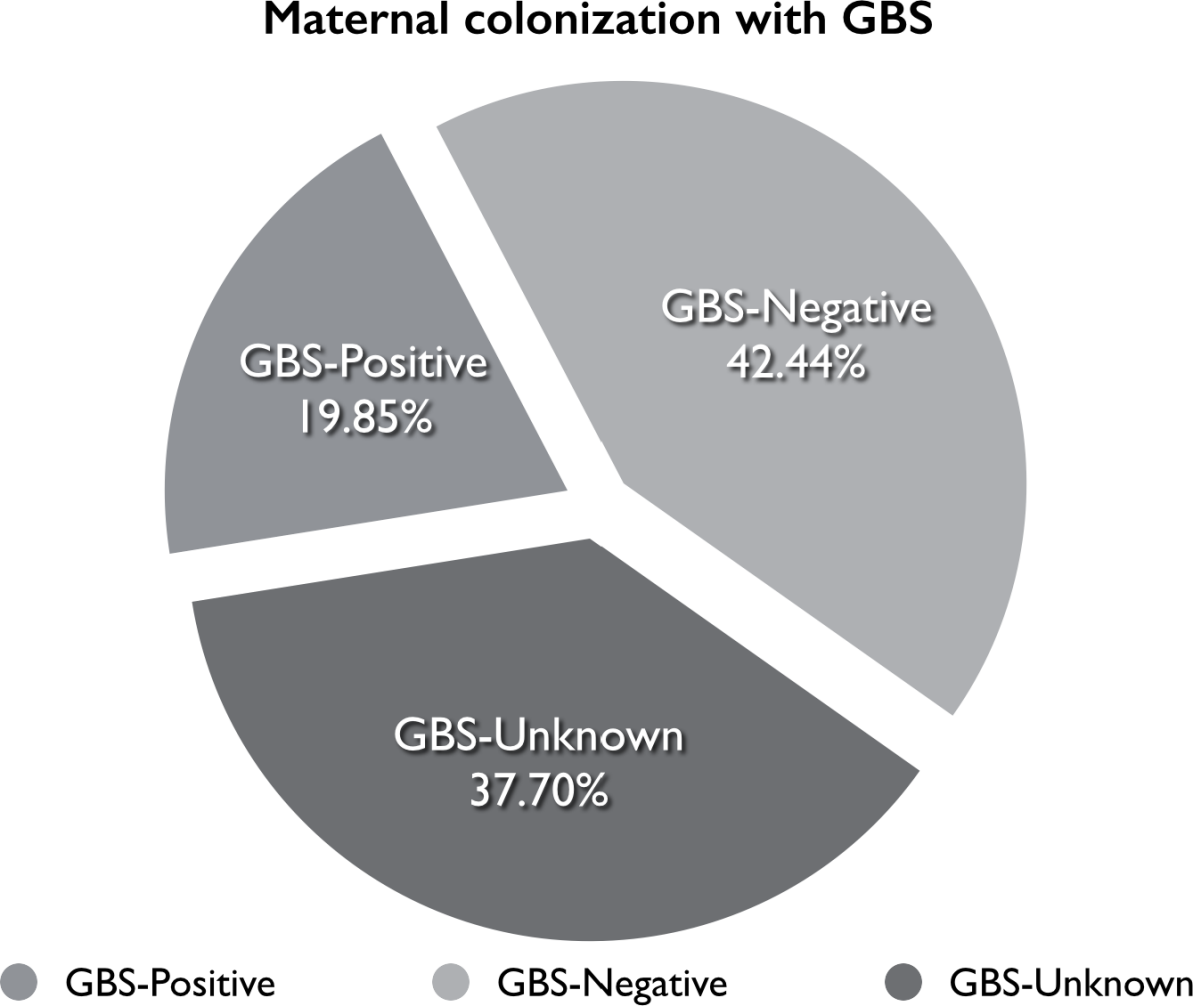
Maternal Colonization with GBS.

Between October 2011 and December 2013, there were 7,681 candidates for vaginal delivery at our institution. The GBS distribution status is presented in Fig 2. During the study period 542 women enrolled and underwent membrane stripping. Of those women, 135 (24.91%) were GBS-screening positive (GBS positive group), 361 (66.60%) were GBS-screening negative (GBS negative group), and 46 (8.49%) did not undergo pre-labor screening for GBS (GBS unknown group).

There were no significant differences between the groups in terms of demographic and obstetric parameters (Table 1). There was a statistically but not clinically significance difference in gestational age at stripping between the groups. Intra-partum characteristics such as simplified Bishop score, induction of labor, fever during labor, analgesia during labor and mode of delivery, were similar among the groups (Table 2).

**Table 1:** Demographic and obstetric characteristics at recruitment.

Characteristics		GBS screening positive group (N = 135)	GBS screening negative group (N = 361)	GBS unknown group (N = 46)	p value
**Maternal age (years)**		30.9 ± 5.4	30.3 ± 4.9	29.5 ± 6.5	0.337
**Gravidity (n)**		3.3 ± 2.4	3.2 ± 2.4	3.3 ± 2.5	0.972
**Parity (n)**		1.9 ± 2	1.7 ± 1.9	1.9 ± 2.1	0.776
**Gestational age at stripping (weeks)**		39.6 ± 1.2	40.0 ± 1.1	39.9 ± 1	0.009
**Simplified Bishop score at stripping** ^**a**^ ^,^ ^**b**^					0.744
	≤ 5	105 (82.0%)	290 (82.2%)	39 (86.7%)	
	> 5	23 (18.0%)	63 (17.8%)	6 (13.3%)	

Values are given as mean ± SD or number (percentage) unless stated otherwise.

a Percentages given are related to available data per characteristic and may differ from total number of patients; Characteristic with more than 5% missing data: Simplified Bishop score at stripping: data available from 128 cases (94.8%) from the GBS screening positive group

b “Simplified Bishop” was calculated according to the article of Laughon SK et al [25].

**Table 2:** Labor characteristics by GBS status.

		GBS screening positive group (N = 135)	GBS screening negative group (N = 361)	GBS unknown group (N = 46)	P value
**Simplified Bishop score at admission to labor** ^**a**^ ^,^ ^**b**^					0.231
	≤ 5	53 (39.6%)	133 (37.0%)	23 (50%)	
	> 5	81 (60.4%)	226 (63.0%)	23 (50%)	
**Initiation of labor** ^**a**^					0.678
	Spontaneous	122 (90.4%)	324 (90.5%)	40 (87.0%)	
	Induction	13 (9.6%)	34 (9.5%)	6 (13.0%)	
**Mode of delivery**					0.597
	Vaginal delivery	109 (80.7%)	284 (78.7%)	34 (73.4%)	
	Instrumental	11 (8.1%)	32 (8.9%)	8 (17.4%)	
	Emergent CD	15 (11.1%)	43 (11.9%)	4 (8.7%)	
	Elective CD	0 (0%)	2 (0.6%)	0 (0%)	
**Epidural anesthesia**					0.242
	Yes	89 (65.9%)	265 (73.4%)	34 (73.9%)	
	No	46 (43.1%)	96 (26.6%)	12 (26.1%)	
**Hours from 4 cm to delivery**		4.9 ± 4.2	5.3 ± 4.9	4.7 ± 3.4	0.362
**Hours from PROM to delivery**		5.3 ± 9.8	4.2 ± 4.9	3.6 ± 6.1	0.263
**Hours from membrane stripping to delivery**		39.9 ± 60.1	42.8 ± 64.6	29.3 ± 44.6	0.370

CD–Cesarean Delivery; PROM–Premature Rupture Of Membranes; GBS–Group B Streptococcus.

Values are given as mean ± SD or number (percentage) unless stated otherwise.

a Percentages given are related to available data per characteristic and may differ from total number of patients; Outcome characteristic with more than 5% missing data: Simplified Bishop score at stripping: data available from 128 cases (94.8%) of the GBS positive group

b “Simplified Bishop” was calculated according to the article of Laughon SK et al [25].

**Fig 3:**
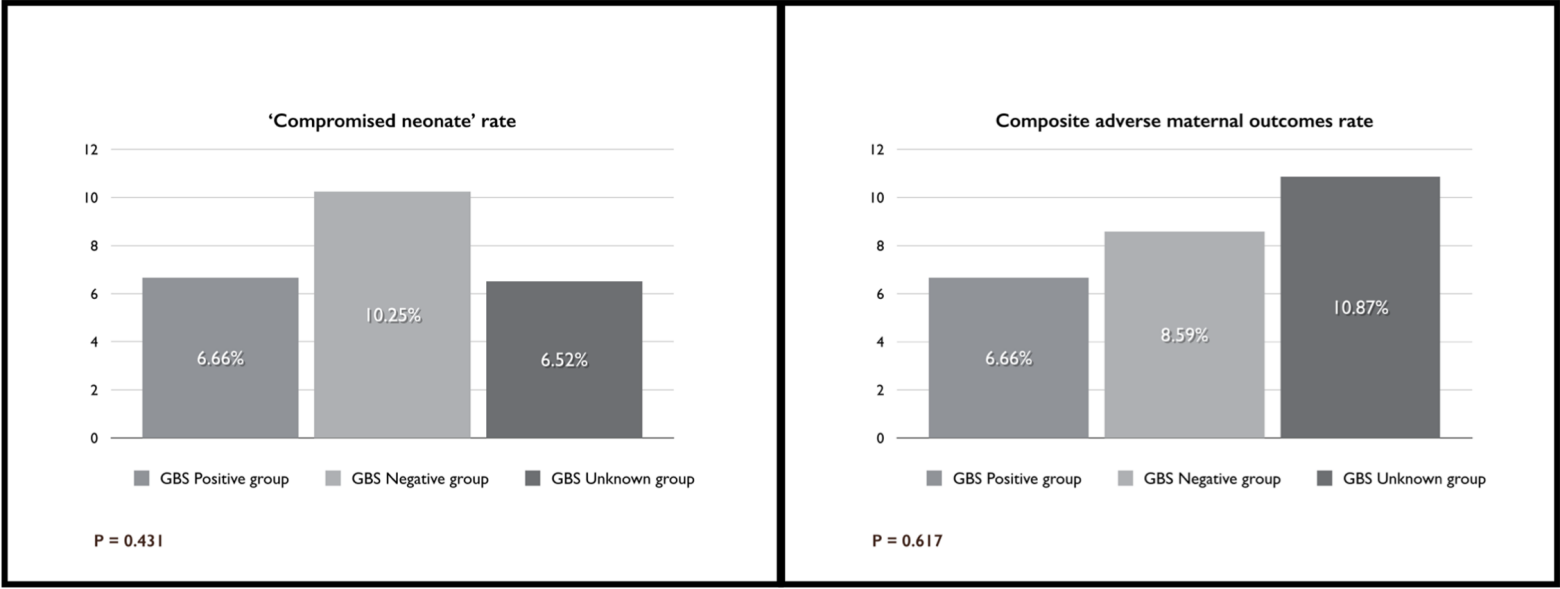
‘Compromised Neonate’ Rate and Composite Adverse Maternal Outcomes Rate.

We observed no cases of neonatal sepsis, death or serious neonatal morbidity in the study. The ‘compromised neonate’ rate was 5.9% (8/135), 8.6% (31/361), and 4.3% (2/46) in the GBS-positive, GBS-negative, and GBS-unknown group, respectively (P = 0.530) (Fig 3). The Odds Ratio between GBS-Positive and GBS-Negative groups was 0.67 [P = 0.33 (95%, CI = 0.30–1.50)]. Neonatal outcomes and the incidence of clinical indicators for possible-early-onset of neonatal infection are summarized in Table 3. The significant difference of hospital stay and NICU admission are due to the GBS-Unknown group.

**Table 3:** Neonatal characteristics and outcomes.

Outcome		GBS screening positive (N = 135)	GBS screening negative (N = 361)	GBS unknown (N = 46)	P value
**Infant gender**					0.741
	Male	69 (51.1%)	198 (54.8%)	24 (52.2%)	
	Female	66 (48.9%)	163 (45.2%)	22 (47.8%)	
**Birth weight, g**		3508 ± 407	3449 ± 429	3422 ± 401	0.305
**Hospital stay of the neonate (days)**		3.2 ± 1	3.0 ± 1.1	3.8 ± 1.3	< 0.001
**NICU admissions**					0.049
	Yes	1 (0.7%)	0 (0%)	1 (2.2%)	
	No	134 (99.3%)	361 (100%)	45 (97.8%)	
**Incidence of clinical indicators for possible-early-onset neonatal infection** ^**a**^					
	1 indicator	23 (17.0%)	59 (16.3%)	8 (17.4%)	
	2 indicators	5 (3.7%)	18 (5.0%)	2 (4.3%)	
	3 indicators	2 (1.5%)	8 (2.2%)	0	
	4 indicators	0	1 (0.03%)	0	
	1 Red flag + 1 indicator	0	1 (0.03%)	0	
	1 Red flag + 2 indicators	0	1 (0.03%)	0	
	2 Red flags only	0	1 (0.03%)	0	
	2 Red flags + 2 indicators	1 (0.7%)	0	0	
	2 Red flags + 4 indicators	0	1 (0.003%)		
**Compromised neonate** ^**b**^		8 (5.9%)	31 (8.6%)	2 (4.3%)	0.530

NICU–Neonatal Intensive Care Unit.

Values are given as mean ± SD or number (percentage) unless stated otherwise.

a Incidence of clinical indicators for possible-early-onset neonatal infection in relation to GBS status (according to NICE guidelines) [20].

b ‘Compromised neonate’ defined as the presence of at least one ‘red flag’ or two or more clinical indicators of possible early-onset neonatal infection [20].

Ninety-four women (69.63%) from the GBS-screening positive group received adequate antibiotic treatment, defined as IAP at least four hours prior to delivery, while forty-one women (30.37%) received inadequate antibiotic treatment. In a subgroup analysis, when stratified by IAP adequacy in the GBS-screening positive group, the risk for ‘compromised neonate was 5.3% (5/94) and 7.3% (3/41) for adequate and inadequate IAP treatment, respectively (P = 0.651).

There were no significant differences among the groups in the frequency of adverse maternal outcomes (Table 4). The composite adverse maternal outcomes rate was 6.66% (9/135), 8.59% (31/361), and 10.87% (5/46) in the GBS-positive, GBS-negative, and GBS-unknown groups, respectively (P = 0.617) (Fig 3). The Odds Ratio between GBS-Positive and GBS-Negative groups was 0.76 [P = 0.46 (95%, CI = 0.35–1.64)]. There were no cases of maternal death or serious maternal morbidity in our study.

**Table 4:** Maternal outcomes.

Outcome		GBS screening positive group (N = 135)	GBS screening negative group (N = 361)	GBS unknown group (N = 46)	P value
**Intrapartum fever**		3 (2.2%)	12 (3.3%)	1 (2.2%)	0.916
**Postpartum fever**		1 (0.7%)	6 (1.7%)	1 (2.2%)	0.501
**Urinary tract infection**		3 (2.2%)	8 (2.2%)	1 (2.2%)	1
**Prolonged hospital stay (≥ 7 days)**		2 (1.5%)	15 (4.2%)	2 (4.3%)	0.309
**Maternal sepsis**		0	0	0	1
**Allergic reactions to antibiotics**		0	0	0	1
**Vaginal laceration**					0.396
	No laceration	90 (66.7%)	231 (64.0%)	29 (63.0%)	
	1^st^ & 2^nd^ Grade	44 (32.6%)	129 (35.7%)	16 (34.8%)	
	3^rd^ & 4^th^ Grade	1 (0.7%)	1 (0.3%)	1 (2.2%)	

Values are given as mean ± SD or number (percentage) unless stated otherwise.

## Discussion

### Main findings

We performed a prospective cohort study to evaluate the effect of antepartum membrane stripping in GBS carriers on adverse perinatal outcomes. In this cohort study, adverse neonatal and maternal outcomes did not differ between GBS-Positive patients and others in the study population undergo membrane stripping.

### Strengths and Limitations

A systematic review of 2,797 women in 22 randomized trials found that in comparison to women without membrane stripping, the risk of maternal and neonatal infection does not increase after stripping of the membranes [10], however this review did not analyze the outcomes by GBS carrier state which comprised 19–26% of all pregnancies. Given the rare neonatal morbidity following membrane stripping, only a few studies have addressed the safety of this procedure in known carriers of GBS. One randomized controlled trial presented in poster form evaluated GBS colonization associated with stripping of the membrane, and concluded that the procedure does not increase maternal colonization [15]. Another randomized controlled trial by Hill et al [21], reported no significant difference in the rate of chorioamnionitis in GBS-Positive patients who underwent stripping versus the non-stripping group, however the numbers in this subgroup were limited. After a thorough search of the literature using the keywords "Group B streptococcus”, “Streptococcus agalactiae”, “GBS in pregnancy”, “Membrane sweeping” and “Membrane stripping”, we were unable to find any prospective, retrospective, or controlled data to suggest that stripping of the membranes in GBS colonized patients is associated with an increased risk of maternal or neonatal infection.

While we performed this study to examine whether membrane stripping increased the risk of neonatal complications in GBS positive women, we found a non-significant risk reduction. This may represent no difference or possibly a protective effect of membrane stripping in an appropriately sized sample. One hypothesis for the possible protective effect may be related to the intrapartum antibiotic prophylaxis in the GBS positive group. Another explanation for the safety of membrane stripping in GBS carriers may be related to the assumed protective effect of the amniotic sac, which provides a barrier between the fetus and the external environment during membrane stripping

In this study the GBS-unknown group had similar maternal and neonatal infection rate as the other groups. This interesting group of patients, in the absence of clinical intra-partum indication for IAP, would not generally receive antibiotic during labor and delivery. If membrane stripping would have jeopardized mothers and neonates by increasing the risk of infection, this group would potentially be the most vulnerable, since they do not receive any antibiotic protection at all. However, the fact that they did not experience a higher rate of adverse outcomes may support our conclusion that membrane stripping is a safe procedure for the mother and neonate.

Some physicians may choose not to strip the membranes in GBS colonized patients because of the concern of fast labor associated with stripping and inadequate antibiotic prophylaxis. As part of the “STRIP-G Study”, we conducted another study comparing adequate antibiotic prophylaxis between GBS carriers who did and did not undergo membrane stripping. In that study we found that antepartum membrane stripping in GBS carriers did not affect the adequacy of antibiotic prophylaxis for GBS [22].

Since the incidence of GBS sepsis is very low in our population, it is practically impossible to perform a study with sufficient significance for analyzing this rare event as a primary end point in one center. Therefore, in this study we used a surrogate endpoint based on the NICE Guidelines of clinical indicators of possible early-onset neonatal infection aiming to detect the “compromised neonate” who are at risk for sepsis [20]. Our inability to report on the GBS status of the neonate is a weakness of this study, however since IAP masks the presence of GBS, it is reasonable to assess these cases of “probable early GBS infection in a neonate” (defined as symptoms and signs of infection in a neonate born to a GBS positive mother, and bacterial cultures from obtained from the neonate that were negative for GBS) as an end-point [23, 24].

Unfortunately the assessment of the neonate could not be blinded to the GBS status of the mother, as the information was included in the medical records. On the other hand, we used a formal structured and validated scale to assess the neonatal outcomes [20].

Although this is the largest study to date following GBS-Positive women after membrane stripping, sample size was not sufficient to establish formal non-inferiority. Furthermore, prior to performing a non-inferiority trial, obstetricians would need to form a consensus regarding a negligible clinical difference in the relevant study outcomes. Differences in population and practice may limit generalizability of our results in other countries. Thus, a large multicenter randomized controlled trial in a heavy colonized population would be useful to confirm our conclusions.

## Conclusion

Membrane stripping is a common and simple obstetrical procedure that may positively influence the transition from pregnancy to the onset of labor, increasing the chance of spontaneous labor and reducing the need for formal induction of labor and the incidence of post-term pregnancy. Its utility is emphasized especially in situations in which our ability to hasten delivery is limited (i.e. grandmultiparity or previous cesarean delivery). Our study and the literature to date shows that antepartum membrane stripping in GBS carriers is not associated with increased maternal or neonatal adverse outcomes as compared to non-carriers. We believe that this study is of clinical relevance with wide application for obstetricians in the community as well as in hospital facilities, providing reassurance and expanding treatment options for GBS carriers.
